# Factors related to the adoption of the Brazilian National Immunization Program Information System

**DOI:** 10.1186/s12913-020-05631-6

**Published:** 2020-08-17

**Authors:** Valéria Conceição de Oliveira, Eliete Albano de Azevedo Guimarães, Gilberto Perez, Fabiana Costa Machado Zacharias, Ricardo Bezerra Cavalcante, Tarcísio Laerte Gontijo, Humberto Ferreira de Oliveira Quites, Gabriela Gonçalves Amaral, Brener Santos Silva, Ione Carvalho Pinto

**Affiliations:** 1 Federal University of São João del-Rei (UFSJ), Midwest Campus, Divinópolis, MG Brazil; 2 Mackenzie Presbiterian University (UPM), Campus Higienópolis, São Paulo, SP Brazil; 3 Ribeirão Preto College of Nursing at the University of São Paulo (USP), Ribeirão Preto, SP Brazil; 4 Associate Teacher of the Nursing Course, Federal University of Juiz de Fora (UFJF), Campus Juiz de Fora, Juiz de Fora (MG), Brazil; 5 Ribeirão Preto College of Nursing (USP) at the University of São Paulo (USP), Ribeirão Preto, SP Brazil

**Keywords:** Diffusion of innovations, Health information systems, Immunization, Primary health care, Nursing

## Abstract

**Background:**

One way to optimize the adoption and use of technological innovations is to understand how those involved perceive, assess and decide to use them. This study aims to analyze the attributes that influence the adoption and use of the Brazilian National Immunization Program Information System (NIPIS) from the perspective of vaccination room workers.

**Methods:**

This is a mixed method research, and a quantitative cross-sectional analytical study, with concomitant triangulation of data, carried out in a region of Brazil by using the Diffusion of Innovation Theory. We used a questionnaire with 183 nursing professionals who work at vaccination rooms in 12 municipalities. To test the research model, partial least squares structural equation modeling (PLS-SEM) and SmartPLS 2.3.0 have been applied to estimate the model. The qualitative research had a descriptive-exploratory character, using interviews (*n* = 18) analyzed through thematic analysis.

**Results:**

The model proposed showed a mean correlation between the perceived attributes in the adoption and use of NIPIS. The results of the multiple regression indicated that the attributes “relative advantage” and “image” have a significant effect at 5% level (T > 1.97), positively influence the adoption and use of NIPIS; the attribute “voluntary use” negatively influences the adoption and use of the system; the attributes “experimentation”, “compatibility”, “profitability”, and “ease of use” did not influence the adoption and use of NIPIS. Emphasis has been placed on aspects that weaken the adoption and use of NIPIS such as lack of good quality internet and resistance to use the technology by some professionals. Workers perceive the importance of NIPIS for the municipality and point out that technological innovation provides data at an individual level, inserted in real time, which makes it possible to assess vaccination coverage. Lack of an unstable internet compromises data release due to system slowness.

**Conclusions:**

The mixed method allowed an in-depth analysis of the adoption and use of NIPIS in the Western Health Macroregion of Minas Gerais State, and similarities were observed in the results. The attribute “relative advantage” is the one that most influences the adoption and use of NIPIS, which is the strongest predictor of innovation adoption rate.

## Background

The United States Centers for Disease Control and Prevention (CDC) defines computerized databases as computerized immunization systems (IIS) that collect and consolidate the doses of vaccines administered to people living within a given geopolitical area [1]. They are instruments for assessing and monitoring immunization programs, and can have a huge impact on vaccination coverage rates [2, 3]. Its use is associated with a reduction in dropout rates, a reliable analysis of vaccination coverage, in addition to increasing the opportunity for vaccination [4, 5].

Over the last 16 years, the Brazilian National Immunization Program (NIP) has been using information systems with aggregated data. Although these systems are adequate for vaccination coverage assessment, they do not allow information assessment such as doses of vaccines administered in individuals and their place of residence [6]. Thus, they become an insufficient technology in face of the new demands of immunization programs [7]. In 2010 a NIP nominal information system, called the Brazilian National Immunization Program Information System (NIPIS), with individual data entry and by origin, was developed by the Department of Informatics of the Brazilian Health System (*Departamento de Informática do Sistema Único de Saúde,* abbreviated DATASUS) [8].

Such technological innovation allows follow-up of individuals vaccinated in several places in Brazil [8]. Also, it allows to assess coverage with better accuracy; to identify individuals vaccinated; to provide data on adverse events following immunization (AEFI); to control the validity of immunobiological agents [8–10]; and to improve decision-making against vaccination activities [10].

The Ministry of Health faces challenges for the implementation of the system since it was introduced [8]. A study aiming to assess the implementation of NIPIS in Brazilian municipalities pointed out a partial performance of the system, signaling problems in its use by the nursing team that works in vaccination rooms. Even with the implementation of NIPIS web, paper forms are still kept to record vaccination activities, which presupposes flaws in their use [11].

Successful implementation and use of technological innovation such as NIPIS are generally related to adaptation and several factors that go beyond the adequacy of the organizational environment such as users, their qualification and acceptance of innovation. They must be approached in an innovative way, according to the specific needs of each system and each user group [12].

Adopting an innovation depends directly on how it is conveyed to future adopters. The Diffusion of Innovation Theory (DIT) defines an innovation as an idea, practice or object that is perceived as new by an individual [13]. Innovation must have attributes in order for people to be attracted to it [13]. Moreover, diffusion of innovation is crucial and determinant for its adoption or rejection by individuals/adopters, since it implies structural and process changes in the daily life of health professionals and service users [14]. This process starts individually based on the knowledge of innovation, which occurs in the implementation process, and culminates in its adoption and use [14].

Implementation of changes in the work process, structure and behavior of a health organization can be considered one of the most difficult and challenging tasks when carrying out an innovation project [15]. The way in which a technological innovation is adopted depends on the attributes/characteristics of usage perceived by users [8, 16].

It is necessary to analyze the attributes that have influenced the adoption and use of NIPIS among vaccination room workers, because a way to optimize the adoption and use of technological innovations is to understand how those involved perceive, assess and decide to use them [17].

In this sense, this study aims to analyze the attributes that influence the adoption and use of NIPIS from the perspective of vaccination room workers.

## Methods

This is a mixed method study with concurrent data triangulation. This research enables simultaneous collection of quantitative and qualitative data in order to identify convergences, differences or combinations between them, at the time of data analysis [18]. Qualitative data were incorporated to better understand the factors of the organizational context of the municipalities that can influence the attributes of DIT in the process of adopting and using NIPIS.

This study was developed in the Western Health Macroregion of Minas Gerais State, which has a large territorial extension (31,543 km^2^), a high Human Development Index (HDI) and a diversified economy. It covers about 1,289,538 inhabitants and 55 municipalities, with 46.3% of small municipalities (under 10,000 inhabitants), 38.9% of medium-sized municipalities (10,000 to 50,000 inhabitants), and only 14.8% of large municipalities (over 50,000 inhabitants) [19]. Concerning Family Health Strategy coverage (FHS), considered a model of primary health care in Brazil, 89.6% of municipalities have a high coverage. The region has 261 vaccination rooms [20], and in 2018 1,090,148 doses of vaccines were administered [8].

For data collection, vaccination rooms in 12 municipalities in the region were selected, covering 87 vaccination rooms. The selection of municipalities, which was the object of this research, was based on a preliminary study with a quantitative approach, carried out in 55 municipalities to assess the degree of implementation of NIPIS [11]. The degree of implementation was defined by a scoring system with different weights for established and validated criteria, according to the level of importance observed. The degree of implementation was divided into adequate (80 to 100%), partially adequate (60 to 79.9%), inadequate (40 to 59.9%), and critical (under 40%). It is important to note that no municipality has achieved adequate implementation [11].

For this study we chose to intentionally select the four municipalities with the largest number of vaccination rooms for each degree of implementation of NIPIS established (partially adequate, inadequate and critical). When there were two or more municipalities with the same number of rooms, we selected the one that was closer to the research institution. Quantitative and qualitative data were collected simultaneously between June and August 2018.

This is a quantitative, cross-sectional and analytical study. The study involved 183 nursing professionals (nurses and nursing technicians) who carried out vaccination activities and who were present at the health unit at the time of data collection. In Brazil, the nursing team is in charge of vaccination activities in the public health service; for this reason, nurses and nursing technicians were selected as research participants. The inclusion criterion was working time in the vaccination room for more than 6 months.

We used the questionnaire validated [21]. It was adapted and validated in Brazil [16]. The researchers applied personally the questionnaire in a private room of each health unit after participants were informed about the research project and signed the Informed Consent Form (ICF). For this study, in addition to the 27 questions of the instrument, three questions were included. A 10-point scale was adopted for assessment; 1 point indicated the lowest degree of importance, and 10 points indicated the highest. The questions included refer to the attributes “visibility” (V29) and “voluntary use” (V28 and V30). The perceived attributes of an innovation [13] and its respective variables in the instrument are described in Table 1, and were used in the research model.

**Table 1:** Perceived attributes of an innovation and its respective variables

Attributes	Description	Tool variables
Relative advantage	The degree by which an innovation is perceived as better than its precursor.	V2, V5, V8, V13, V25
Compatibility	The degree by which an innovation is perceived as consistent with the values, past experiences and needs of adopters.	V9, V21, V23
Experimentation	The degree by which an innovation can be experienced before its adoption.	V3, V12, V24
Ease of use	The degree by which an innovation is perceived as easy of use.	V6, V14, V20, V22
Image	The degree by which the use of an innovation is perceived to improve an individual’s image or the status of a social system.	V4, V11, V27
Volutary use	The degree by which the use of an innovation is perceived as voluntary or spontaneous.	V1, V18, V28, V30
Visibility	The degree by which an innovation becomes visible to the individuals or groups of an organization.	V10, V16, V19, V29
Profitability	The degree by which the results of using an innovation are tangible.	V7, V15, V17, V26

*DIT* Diffusion of Innovation Theory

**Source:** created by the authors.

**Fig. 1:**
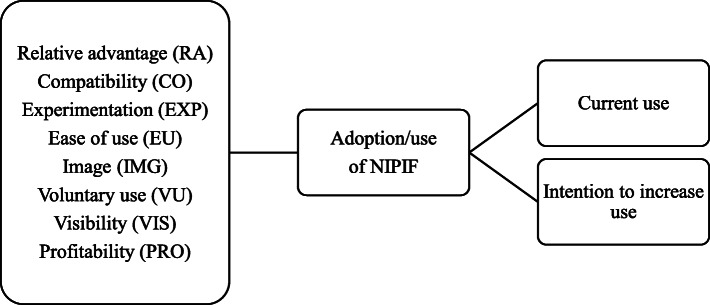
Search model

Figure 1 represents the proposed model for the research. The Independent Variables (IV) were represented by the set of eight attributes described in Table 1. It is observed that the adoption and use of NIPIS is directly affected by the attributes perceived. The Dependent Variable (DV) is the “adoption and use of NIPIS” by vaccination room workers. DV was measured based on the statement made by the professional during data collection regarding the current level of NIPIS use and the statement to intensify its use.

To test the research model, partial least squares structural equation modeling (PLS-PM) was applied [22], and SmartPLS 2.3.0 [23] was used to estimate the model. Variables with a correlation ≥ to 0.70 were acknowledged for the purpose of attribute “reliability” [22].

We assessed the load of each variable observed in its respective attributes; we verified the Average Variance Extracted (AVE); and we assessed composite reliability values for the attributes and the variable “adoption and use of NIPIS”. Subsequently, a structural model validation was performed by verifying the values of the coefficient of determination of variance R^2^ and Student’s t-test. We used the SmartPLS bootstrapping algorithm, with parameter 500 for the number of cases and samples to obtain the results of the Student’s t-test distribution [23]. The Student’s t-test results depend on the number of questionnaires answered. The Student’s t-test distribution value was 1.97, 95% confidence interval and 0.05 significance for a sample of 183 interviewees (degrees of freedom) [22].

The qualitative research was descriptive and exploratory. For data collection, in order to deepen the knowledge about the factors of the organizational context, under the influence of the DIT attributes, the agents of change of the 12 municipalities were listed for the interview. According to DIT, the change agent is acknowledged by technical knowledge and must be able to influence the consumers of innovation [14]. Managers and technicians involved with the implementation of NIPIS in the municipalities were selected. In most municipalities, the system change agent was the professional designated to be a Technical Reference (TR) in immunization. However, in some municipalities, the Primary Health Care Coordinator was identified as a change agent, in addition to the TR in immunization, the FHS nurse, and the Municipal Health Secretary. We interviewed the professional in charge of implementing NIPIS in the Western Health Macroregion and the reference professional in Minas Gerais State, which totaled 18 participants. All those selected as change agents agreed to participate in the research and signed the ICF.

It was used an individual interview with a semi-structured script, addressing guiding questions regarding the theory attributes. The interviews took place at the professional’s own workplace and were recorded, transcribed in full, with an average duration of 15 min. The interviewees were coded with letter I, followed by the chronological sequence of the interviews, to maintain participant anonymity.

Data analysis occurred through pre-analysis, exploration of the material and treatment of results, and inference and interpretation [24]. The attributes of the theory were used as thematic categories. Quantitative and qualitative data were analyzed separately and integrated into the level of interpretation.

The Human Research Ethics Committee of *Escola de Enfermagem de Ribeirão Preto* of *Universidade de São Paulo* (EERP/USP) approved this research, under Opinion 2768.82.

## Results

The socioprofessional profile characteristics of the study participants are in Table 2. Most of vaccination room workers are female (94%) and have 9 years of experience in Primary Health Care. Only 37.7% have graduate degrees and almost half (48.1%) had a temporary employment contract. As for technology use at work, 95.1% of interviewees reported that they have easy access to the computer and that they use it a lot (73.8%). NIPIS Web is in 70.5% of vaccination rooms.

**Table 2:** Socioprofessional profile of vaccination room workers, Minas Gerais, Brazil, 2018

Variables	n (%)	AM^a^ SD^b^
**Age**	–	39.1 ± 8.77
**Gender**		
Female	172 (94%)	
Male	11 (6%)	
**Professional category**		
Nurse	85 (46.4%)	
Nursing technician	75 (41.1%)	
Nursing assistant	20 (10.9%)	
Community Health Agent	3 (1.6%)	
**Education**		
High school	103 (56.3%)	
Higher education	80 (43.7%)	
**Graduation degree**		
Undergraduate	11 (13.8)	
Specialist	67 (83.8%)	
Master	2 (2.4%)	
**Employment relationship**		
Public servant	95 (51.9%)	
Hired	88 (48.1%)	
**Working time at PHC**	–	9.6 ± 7.77
**Computer use**		
Yes	183 (100%)	
**Internet use**		
Yes	177 (96.7%)	
No	6 (3.3%)	
**Classification of computer use**		
Fairly	135 (73.8%)	
Roughly	42 (23%)	
Slight	6 (3.3%)	
**Easy access to the computer**		
Yes	174 (95.1%)	
No	9 (4.9%)	
**Type of NIPIS**		
Web	129 (70.5%)	
Desktop	54 (29.5%)	

Source: created by the authors

^a^*AM* arithmetic mean, ^b^*SD* standard deviation

**Fig. 2:**
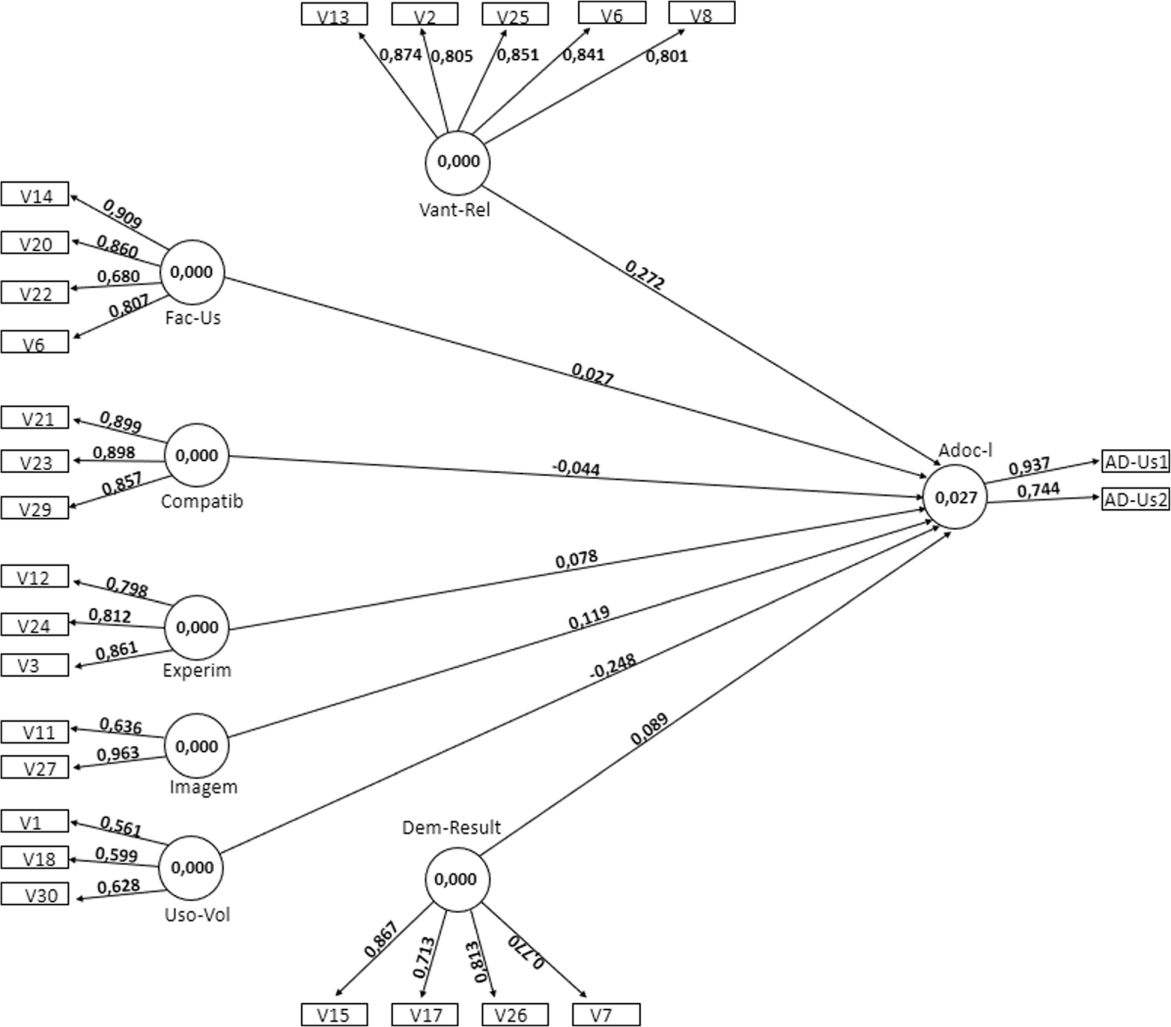
Final model of the search

Most of the observed variables (V1 to V30) revealed correlation values equal to or greater than 0.7, except for variables V4 and V28 referring to “image” and “voluntary use”, respectively. “Visibility” was excluded because it presented values of correlations lower than 0.7 for all its variables. After adjustments, a new processing was performed. The result of the final model is in Fig. 2. The variable V11 of “image”, and variables V1 and V18 of “voluntary use” were included in the final model because they had values close to 0.7 [22], and because they are important in validating the model.

Figure 2 shows standardized regression coefficients associated with each of the attributes, indicating how much they impact on the latent variable “adoption and use” when they increase in unit. “Relative advantage” presents the highest coefficient of regression (0.27) with the variable “adoption and use”. Thus, when “adoption and use” increases by one unit, the greatest contribution to this variation comes from “relative advantage”. On the other hand, “voluntary use” (− 0.25) and “compatibility” (− 0.04) presented negative values.

The other attributes (image, ease of use, profitability, and experimentation) positively affected the adoption and use of NIPIS. Within the circle, which represents the DV “adoption and use”, the R^2^ value obtained was 23%. DIT attributes explain 23% of the adoption and use of NIPIS.

Table 3 presents the main indicators obtained for the adjusted model, without “visibility”. The values obtained for the mean explained variance and the composite reliability were higher than 0.5, as recommended, except for “voluntary use” [25]. Indicators of a specific construct must converge or share a high proportion of variance. Although the “voluntary use” has an AVE of less than 0.5, reliability was certified through Composite Reliability (CR), which is a more robust indicator of precision when compared to Cronbach’s alpha coefficient [22].

**Table 3:** Calculation indicators of the adjusted model; standardized coefficient and T value of the proposed model. Minas Gerais, Brazil, 2018

Attributes	AVE	Composite Reliability	***R***^**2**^	Standardized coefficient	T value > 1.97
Adoption and use	0.7	0.8	0.23	–	–
Compatibility	0.8	0.9		- 0.044	0.503
Results report	0.6	0.9		0.089	0.911
Experimentation	0.7	0.9		0.078	1.924
Ease of use	0.7	0.9		0.027	0.303
Image	0.7	0.8		0.119	3.411
Voluntary use	0.4	0.6		−0.248	4.143
Relative advantage	0.7	0.9		0.272	3.860

Source: created by the authors

The multiple regression results indicated that “relative advantage” and “image” have a significant effect at the 5% level (T > 1.97), i.e., positively influence the adoption and use of NIPIS. “voluntary use” negatively influences the adoption and use of the system (Table 3). “experimentation”, “compatibility”, “results report” and “ease of use” did not influence the adoption and use of NIPIS.

In the qualitative approach, from data analysis, it was possible to interpret the influence of DIT attributes in adopting and using NIPIS.

**Relative advantage**, in the use and adoption of NIPIS, was evidenced by the advantages of using the system to improve vaccine coverage. Participants make comparisons between the paper-based record system used previously and technological innovation. *The advantage I see is that it improved coverage* (I4). *In the past, registry was performed with paper forms, and sometimes it was not visible, but now with registry at NIPIS, its is now visible* (I16). *Any information you need is faster to find in the program than in the notebook. I think it’s a lot better* (I5). *Then, data are filed and we can get access to them in another city, if the child gets vaccinated in another city, the information will be displayed for us* (I1).

Furthermore, interviewees perceive the importance of NIPIS for immunization room care. *It is a benefit for the immunization room, since we now have the data from vaccination online* (I15). *The municipality only benefits from computerizing things, the agility of searching for a given information, a vaccination card, coverage* (I10).

**Image** has been improved in health services, and respondents noticed. In the past we used paper (I10); But, looking back, for frontline workers (Primary Health Care Unit), using NIPIS is fantastic, invaluable (I14). In fact, I think NIPIS was an innovation. It was the best thing they have done for many years (I7).

In **voluntary use**, the statements below exemplify an imposition of higher management spheres in the adoption and use of NIPIS linked to transfer of funds. *Well, it was not exactly a choice. It is a guideline, a decision of the central level of the State Health Office* (I14). *It was a system deployed overnight and we had to move, to use the system now!* (I7). *The municipality that does not register the individuals vaccinated and the flow will lose resources* (I17).

Absence of good internet connectivity and consequently slow system content were acknowledged as detrimental factors for the adoption of NIPIS as well as compromising the work of the team. *The system is very slow. You type the first number, and it is loading; you type the second number, and it is also loading, so it makes things more complicated. This happens mainly here in my unit. Time is short, because the flow of people is very large, sometimes I get in the way of the time I spend typing vaccine at NIPIS* (I1). *It’s because we do not have internet in all immunization rooms* (I2).

**Fig. 3:**
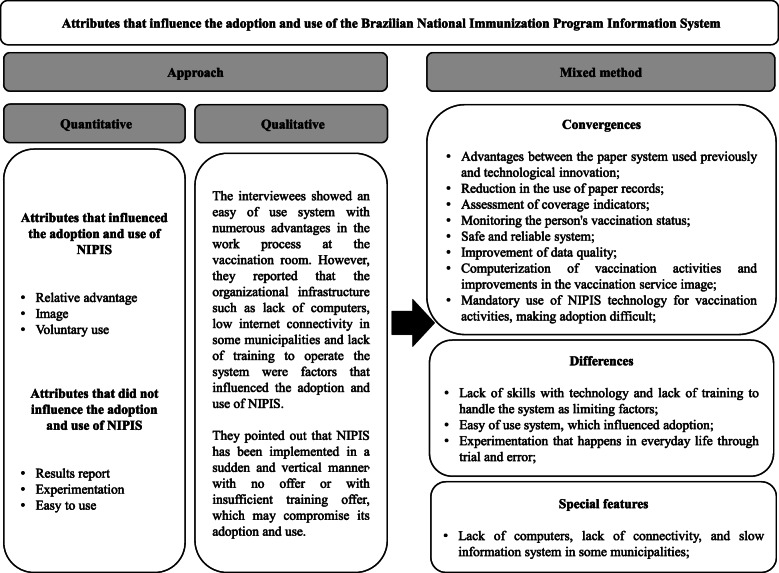
Quantitative and qualitative data integration, Minas Gerais, Brazil, 2018

Figure 3 presents the convergences, divergences and special features of quantitative and qualitative data integration.

## Discussion

The results of the application of the model proposed in the research demonstrated an average correlation [23] among the attributes perceived in the adoption and use of NIPIS (“relative advantage”, “image”, and “voluntary use”). It is important to emphasize that for Rogers [14], the perceived attributes of an innovation are meaningful predictors of use and adoption rate, however other organizational context variables also collaborate for technological innovation usage.

“Relative advantage” was the attribute that most explained the model proposed in the research, corroborating Rogers [14], when affirming that this attribute is the strongest predictor of the rate of adoption of an innovation. Benefits and burdens of an innovation will first be assessed to determine its “relative advantage” [14].

The findings pointed out that NIPIS technological innovation provides individual-level data accessible in real time, which makes it possible to assess vaccine coverage. Similar to NIPIS, the IIS of Denmark, Iceland, Malta, Norway, Portugal (mainland), Spain (Andalusia), Sweden and the United Kingdom (England) [5], and Minnesota [26] also insert vaccine data in real time. The IIS that is not fed properly, with data typing in real time, can compromise the integration of nominal vaccination data and, consequently, the reach of vaccination coverage [3]; in addition to leading to under-registry and/or duplication of records, compromising data quality [27].

The use of IIS, for vaccine coverage analysis, is described in nursing literature and points out advantages such as more accurate calculations, speed and low cost, in addition to allowance of validity of doses analysis and opportunity of vaccination [4, 10, 28]. To guarantee the completeness of vaccine coverage, IIS should, ideally, be filled with data from all places that administer vaccines, whether public or private, covering the entire population, maintaining information on all vaccines recommended by health authorities [5, 29].

However, systems in many countries only capture vaccines provided by public services and recommended by the national vaccination schedule [5], as is the case of the Brazilian IIS [11], the American IIS (California) [30], and the Italian IIS [27]. The disadvantage of such systems is that they may not provide reliable estimates of vaccination coverage since data do not contain complete information about the population vaccinated [30].

If an IIS is accepted, good quality and updated data is useful for vaccinators, and it makes the work easier in the immunization room [2]. Furthermore, a stable internet is necessary to avoid compromising data entry, due to the slowness of the system and, consequently, the maintenance of paper forms. Some Brazilian municipalities face important challenges in implementing NIPIS, including good quality internet access [11] due to the size and diversity of the country.

Nominal registry with NIPIS requires more time for typing and an unstable internet can compromise data entry due to the slowness of the system and, consequently, the maintenance of the old system of paper forms for vaccination data registry. Studies on the implementation of electronic medical records have identified technical limitations such as slow system operation, slow login and combination of slow and network problems that can impact the ease and effectiveness of using the information system [12, 31, 32].

“Voluntary use” negatively influenced the adoption and use of NIPIS. The use of the system was perceived as an imposition of management bodies, with demands for its use linked to transfer of funds. Generally, innovations that require an individual adoption decision are adhered to more quickly than when the innovation decision is made by an organization [14].

In certain locations like Michigan there is a law under which child immunization providers are required to register immunizations within 72 h of administration in the Michigan Care Improvement Registry (MCIR) system. In 2007, 95% of children aged 19 to 35 months had 2 or more immunization records in the MCIR [33].

Also in Minnesota, pharmacists are required to notify all doses of vaccines administered to the Minnesota Immunization Information Connection, a population-based system for the state of Minnesota. This system has been in operation since 2002, and almost 99% of children, 80% of adolescents and 90% of adults have at least 2 immunization records in the system [26].

In contrast, in San Diego, the internet-based IIS is used by public and private organizations and medical systems, which voluntarily report immunization data [30]. The study carried out to test the reliability of the San Diego IIS records, compared to the telephone survey records of 553 patients, identified vaccine coverage in the ISS far below the vaccine coverage of the telephone survey [30].

In this study, workers realized the importance of NIPIS for the municipality as demonstrated in the statistical significance and convergence of the qualitative data of “image” in the adoption and use of NIPIS. It is important to emphasize that the implementation of NIPIS should be followed by continuous assessment to identify barriers and gaps and propose timely solutions. It is necessary to understand the reasons why users accept or reject certain systems in order to later predict, explain and modernize these systems [34]. High potential technological innovation is useless if users, for some reason, do not adopt and use it [34].

Minnesota Immunization Information Connection (MIIC) is considered one of the main IIS in the United States. To maintain system excellence, a continuous quality improvement process is carried out, which involves an interactive discussion, sharing regarding immunization rates and education to use MIIC [26]. In Brazil, important advances have been made, which enhances the adoption and use of NIPIS, such as the expansion of internet access with adequate quality and speed, which allows complete and correct vaccination records, with electronic access in real time in all Brazilian municipalities.

Technological innovation alone is not enough to guarantee the benefits of its adoption and use. It is essential that planners and service managers identify the human and organizational processes involved in the motivation for adoption and use of innovation [15].

As a study limitation, components of diffusion of innovations such as communication channels, time and social system were not considered. Further research may broaden the research model and explore other factors that affect the adoption and use of NIPIS. The choice of change agents, although adequate to the theory used, did not include professionals working at a vaccination room that handles NIPIS in the municipalities.

## Conclusion

The mixed method allowed to analyze in depth the adoption and use of NIPIS in the Western Health Macroregion of Minas Gerais State, and similarities were observed in the results. There was a strong influence of “relative advantage” followed by “image” and a negative influence of “voluntary use”. There were difficulties faced in the process of accepting and using NIPIS.

## Data Availability

Data sets used and/or analyzed during the current study may be made available by the corresponding author upon reasonable request.

## Abbreviations

NIP: Brazilian National Immunization Program
DATASUS: Department of Informatics of the Brazilian Health System
NIPIS: National Immunization Program Information System
DIT: Diffusion of Innovation Theory
HDI: Human Development Index
FHS: Family Health Strategy
IV: Independent Variables
DV: Dependent Variable
RA: Relative Advantage
CO: Compatibility
EXP: Experimentation
EU: Ease of Use
IMG: Image
VU: Voluntary Use
VIS: Visibility
PRO: Profitability
PLS: Partial Least Squares
SEM: Structural Equation Modeling
AVE: Average Variance Extracted
TR: Technical Reference
EERP/USP: *Escola de Enfermagem de Ribeirão Preto* of *Universidade de São Paulo*
CR: Composite Reliability
IIS: Immunization Information System
UFSJ: *Universidade Federal de São João del-Rei*
